# Supplementary material for: MAVS is important for antiviral defense against influenza A virus in a human respiratory epithelium model
Source: PLoS One. 2026 Jun 3;21(6):e0350839. doi: 10.1371/journal.pone.0350839 (PMC13232818; doi:10.1371/journal.pone.0350839)
Supplement: S1 Fig — (PDF) [file pone.0350839.s001.pdf]

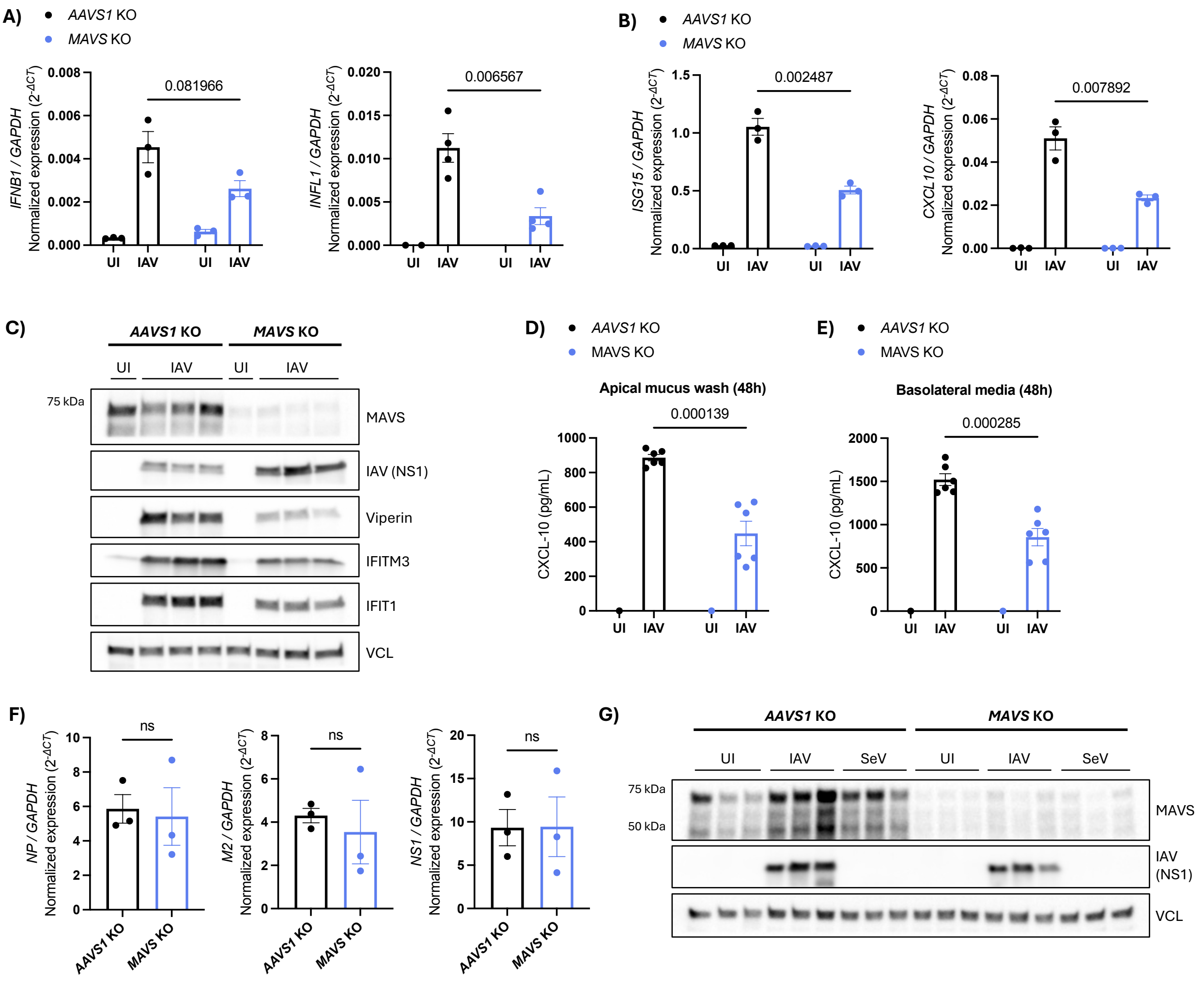

Supplementary data

A) RT-qPCR analysis of *IFNB1* and *IFNL1* in AAVS1 or MAVS KO HAE-ALI cultures infected with IAV (MOI 0.5) or left uninfected for 16 hours. Each data point represents an independent culture (n=3) derived from donor nr. 1. For *IFNL1*, some uninfected cultures had undetectable levels. **B)** RT-qPCR analysis on ISGs in cultures infected with IAV (MOI 0.5) or left uninfected for 16 hours. Each data point represents an independent culture (n=3) derived from donor nr. 1. **C)** Western blot analyzing protein levels of MAVS, various ISGs and viral (NS1) protein in AAVS1 or MAVS KO HAE-ALI cultures 48 h post-infection with IAV. Vinculin (VCL) was used as a loading control. Each lane represents an independent culture (uninfected n=1, infected n=3) derived from the same donor. **D)** hCXCL-10 ELISA performed on the apical mucus wash from AAVS1 or MAVS KO HAE-ALI cultures 48 hours after infection with IAV (MOI 0.5) or left uninfected. Each data point represents an independent culture (uninfected n=1, infected n=6) derived from the same donor. **E)** Basolateral media underneath the transwell membrane from the same experiment as in (D) was also analyzed using a hCXCL-10 ELISA. Each data point represents an independent culture (uninfected n=1, infected n=6) derived from the same donor. **F)** RT-qPCR analysis of viral RNA from three different viral segments from AAVS1 or MAVS KO HAE-ALI cultures infected with IAV (MOI 0.5) for 16 hours. Statistical difference was determined by an unpaired Welch's t-test; "ns" indicates p-value > 0,05. **G)** Western blot analysis of MAVS and viral (NS1) protein levels in AAVS1 or MAVS KO HAE-ALI cultures infected with IAV (MOI 0.5) or SeV (30 HAU/mL) or left uninfected for 16 hours. VCL was used as a loading control. Each lane represents an independent culture (n=3) derived from the same donor.

Bars represent mean ± s.e.m. Unless otherwise stated, statistical differences were determined by multiple unpaired t-tests with FDR correction using the two-stage step-up method of Benjamini, Krieger and Yekuteili (desired FDR = 5%). Q-values are shown above each comparison. Statistical analysis was not performed for uninfected groups (A-B and D-E).
