# Supplementary figures and images for: MAVS is important for antiviral defense against influenza A virus in a human respiratory epithelium model
Source: PLoS One. 2026 Jun 3;21(6):e0350839. doi: 10.1371/journal.pone.0350839 (PMC13232818; doi:10.1371/journal.pone.0350839)

Fig. 1C

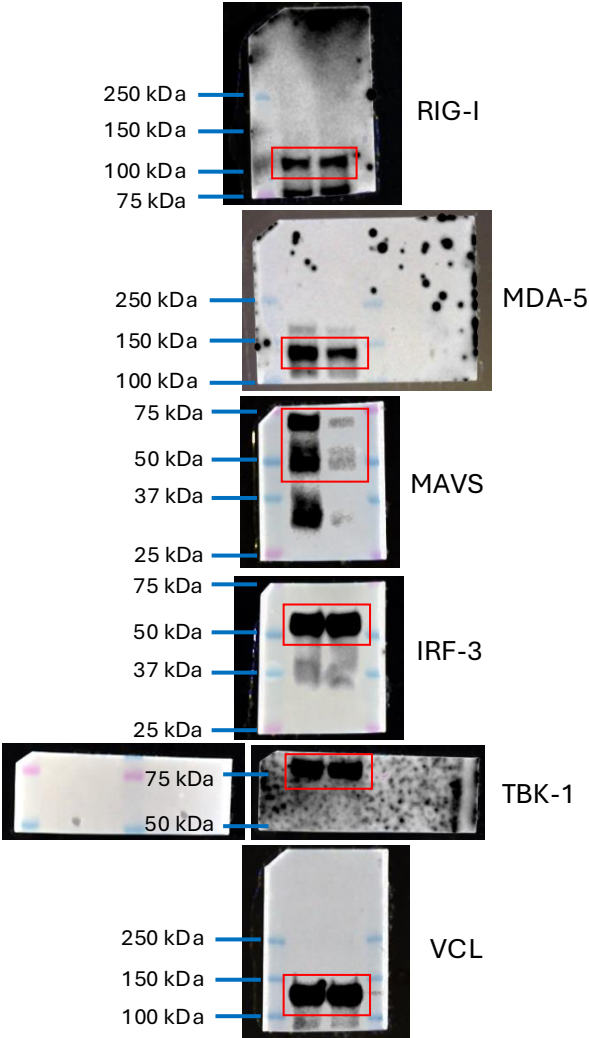

Fig. 1F

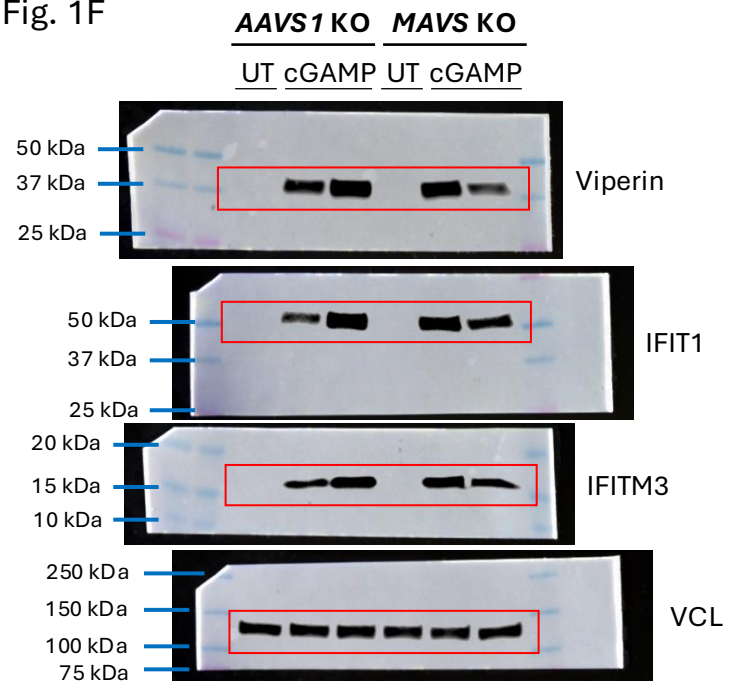

Fig. 2E

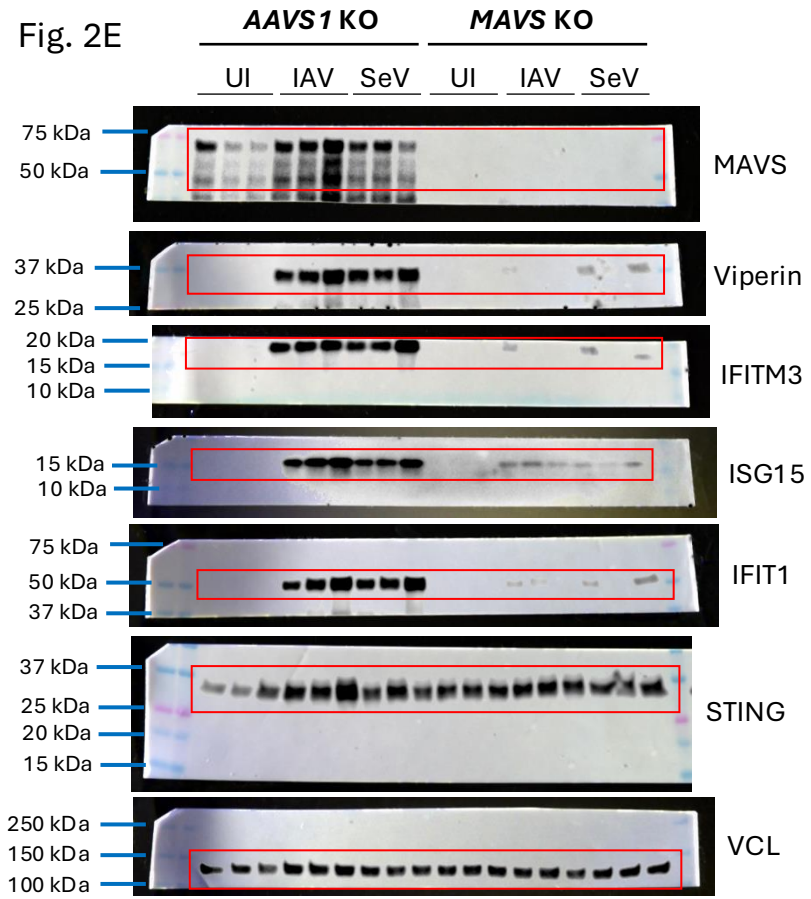

Fig. 3B

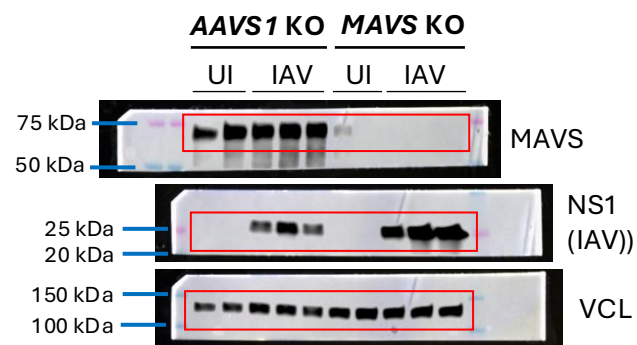

Suppl. Fig. C

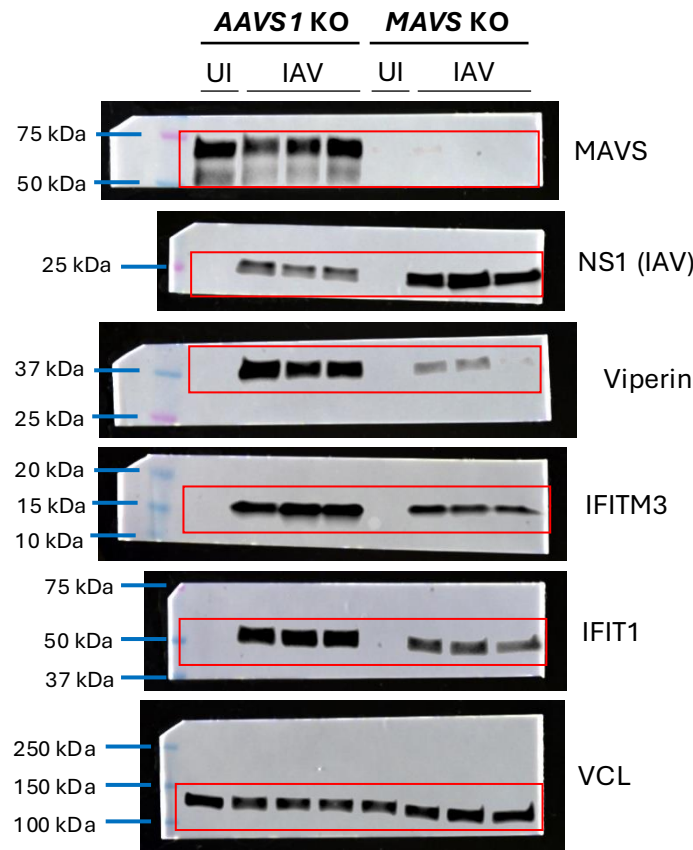

Suppl. Fig. G

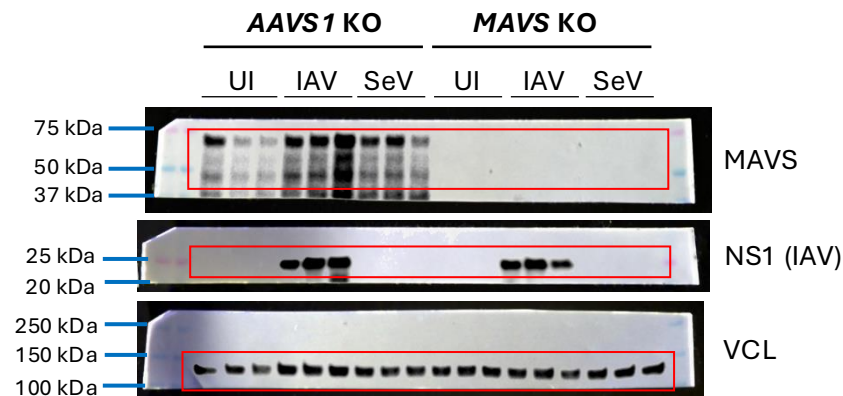

Supplement: S2 Fig — (PDF) [file pone.0350839.s002.pdf]
