# Supplementary material for: MAVS is important for antiviral defense against influenza A virus in a human respiratory epithelium model
Source: PLoS One. 2026 Jun 3;21(6):e0350839. doi: 10.1371/journal.pone.0350839 (PMC13232818; doi:10.1371/journal.pone.0350839)
Supplement: Attachment — Submitted filename: 164518_1_rebuttal_3679824_tb2k67.docx [file pone.0350839.s003.docx]

**Manuscript number:** RC-2025-03340

**Corresponding author(s):** Christian Kanstrup, Holm.

*[Please use this template only if the submitted manuscript should be considered by the affiliate journal as a full revision in response to the points raised by the reviewers.*

*If you wish to submit a preliminary revision with a revision plan, please use our "*[*Revision Plan*](https://docs.google.com/document/u/0/d/1HHp62Tfeb44nd3Gu_3ChB0b8gmMdEog7GjZcZFwHSiQ/edit)*" template.* ***It is important to use the appropriate template to clearly inform the editors of your intentions.****]*

## 1. General Statements [optional]

*This section is optional. Insert here any general statements you wish to make about the goal of the study or about the reviews.*

## 2. Point-by-point description of the revisions

*This section is mandatory.* *Please insert a point-by-point reply describing the revisions that were already carried out and included in the transferred manuscript.*

Reviewer #1 (Evidence, reproducibility and clarity (Required)):

The study by Hemberg et al investigated whether the critical adaptor protein MAVS is required for IFN induction and viral resistance in primary human respiratory epithelial cells infected with influenza A virus (IAV). Specifically, air-liquid interface pulmonary bronchial epithelial cells (ALI-PBECs) are engineered with CRISPR/Cas9 to knockout MAVS (or AAVS1 as a control), followed by IAV infection and subsequent monitoring of the 1) IFN/ISG response and 2) viral replication. The study is thoroughly conducted, the methodology and the results are clearly described, and the study is written in a clear and transparent manner. No overstatements are made and limitations of the study are clearly indicated. I therefore only have a few minor comments:

We would like to thank Reviewer 1 for their assessment of our manuscript. We are pleased with the overall positive comments, especially that the study was considered thoroughly conducted. However, this reviewer also raised some concerns that we will address in the point-to-point review below.

- In the Discussion the following sentence should be nuanced: "However, most studies report no observable effect of MAVS KO on antiviral protection18,20". Please indicate that this refers to the context of IAV infection. I would also clarify that in this sentence you are referring to antiviral protection rather than IFN/ISG induction. (For example: While MAVS is important for IFN/ISG induction upon IAV infection, some studies in MAVS-deficient mice showed that this did not necessarily translate to increased antiviral protection). I would also advice to stay away from 'most studies report no observable effect' when you only reference two publications.

This is a very valid point. We have addressed the comment by adjusting the sentence accordingly:

“The importance of MAVS in IAV infections has only been investigated in few studies using MAVS-deficient mice. While these studies show that MAVS is essential for the induction of IFNs and ISGs upon IAV infection, supporting the findings of the current study, these findings did not translate to increased antiviral protection in the mice18,20.” in the revised version of our manuscript (page 4, line 34-37).

- Fig. 2A and 2B (and 2C and 2D): please indicate the time points in the graphs to better reflect (visually) the difference between the panels.

We agree with this comment by the reviewer. Figure 2A-D have been adjusted to include the infectious time points.

Reviewer #1 (Significance (Required)):

While the results of the study are not surprising (reduced IFN/ISG responses and increased IAV replication in MAVS KO cells), the scientific advance in this study is largely of a methodological nature: the role of MAVS in IAV infection has not been previously studied in this advanced culture model system. The study is of relevance to researchers that are in the immediate research field (virologists, RNA sensing). The study provides a useful example of CRISRP/Cas9 engineering in a complex model system to investigate the role of a particular gene in antiviral innate responses, which could easily be extrapolated to other genes.

We are thankful, that Reviewer 1 appreciates the significance and potential of the complex model used in this study.

Reviewer #2 (Evidence, reproducibility and clarity (Required)):

Review of manuscript RC-2025-03340:

The authors of the manuscript "MAVS is Important for Antiviral Defense Against Influenza A Virus in a Human Respiratory Epithelium Model" present their recent work evaluating innate immune responses in a human cell line. Specifically, they infect primary human respiratory epithelial cells with influenza A virus and use CRISPR-Cas9 to knockout MAVS and demonstrate the importance of this adaptor molecule on type I and type III IFN induction. Their work shows that MAVS is important for restricting viral replication and they conclude that it is a non-redundant protector of human respiratory epithelial cells. Below are comments that the authors should consider during revision of this manuscript.

We thank Reviewer 2 for their careful review of our manuscript. Reviewer 2 provided several valuable suggestions for improvements, which we will address below.

1. The authors use the IAV A/PR/8/34, which is a good, laboratory strain for initial characterization. However, since this model strives to evaluate human cell interactions the authors are encouraged to use a relevant primary human influenza virus isolate in their model. This is important as the host:pathogen interactions may differ significantly when using a human isolate.

This is an important point. We agree that stain-specific differences might affect host-pathogen interactions. Although IAV A/PR/8/34 is well-characterized and easily reproducible in mechanistic studies, it is a laboratory adapted strain. Therefore, we have now added a statement in Discussion acknowledging this limitation:

“An important consideration of this study is the use of the laboratory-adapted influenza A/PR/8/34 (H1N1) strain. While this strain was selected due to its widespread use and ability to provide a robust and reproducible infection model, it has undergone extensive laboratory adaption. Consequently, the host-pathogen interactions observed in this study might differ from those elicited by primary clinical isolates. Validation of these results using contemporary human influenza stains will be important to determine their broader applicability.” (page 5, line 22-27).

2. It was unclear why the authors used SeV for a control.

We thank the reviewer for this important comment. Sendai virus (SeV) was used as a positive control due to its well-established ability to effectively activate RIG-I-dependent antiviral signaling. SeV is widely used as a potent inducer of RIG-I-mediated innate immune responses and provide a strong and reproducible stimulus independent of influenza-specific evasion mechanisms. To clarify this rationale, we have expanded the motivation for using SeV in the manuscript as indicated below:

“To validate disruption of MAVS-dependent signaling, MAVS KO HAE-ALI cultures were infected with Sendai virus (SeV). SeV is a well characterized activator of the RIG-I pathway and is widely used as a positive control in studies of antiviral innate immunity. Infection with SeV induces a strong and reproducible activation of downstream signaling, thereby allowing validation of pathway integrity.” (page 2, line 44-46 and page 3, line 1-2).

Reviewer #2 (Significance (Required)):

A. Strengths of this study are the model developed and the evaluation of the MAVS component within primary human epithelial cells. Limitations include the use of a lab strain of influenza that is mouse-adapted (minimizes potential interaction with human host intracellular machinery), lack of a uniform knockout, and the inclusion of SeV without indicating the benefit of using this virus as a control.

B. The advance in this publication is modest as it uses a non-uniform knockout model to demonstrate that an already-appreciated mechanism is involved in host:pathogen interactions. Also, without the use of a human virus isolate it is unclear if this is applicable beyond the lab setting.

C. The audience for this is basic research.

D. I am a basic science researcher who studies host:pathogen interactions using influenza models of vaccination, virus pathogenesis, and secondary bacterial infections.

We would like to thank Reviewer 2 for their time and helpful comments. We have addressed their concerns to the best of our ability. We are pleased that the reviewer recognized the strength of our model in assessing the role of MAVS in primary respiratory epithelium.

Reviewer #3 (Evidence, reproducibility and clarity (Required)):

The manuscript "MAVS is Important for Antiviral Defense Against Influenza A Virus in a Human Respiratory Epithelium Model" by Hemberg et al. demonstrates that reduction of MAVS expression in human respiratory epithelium leads to diminished induction of the type I IFN gene IFNB1, the type III IFN gene IFNL1, and multiple interferon-stimulated genes in response to IAV infection. They further show increased viral replication at 48 hours post-infection in MAVS-reduced epithelial cultures, as evidenced by elevated viral gene (NP, M2, NS1) transcripts and viral protein (NS1) levels.

We would like to thank Reviewer 3 for their assessment of our manuscript. That reviewer raised some major and minor concerns that we will address in the point-to-point review below.

Major comments:
The authors convincingly show reduced MAVS expression in their CRISPR-Cas9 KO epithelial cultures compared to AAVS1 KO controls. However, residual MAVS expression remains detectable by western blot (Fig. 1C). No data is provided regarding cell-type specificity of the gene knockdown, although immunostaining and transcript analyses confirm the presence of both ciliated and secretory cells in MAVS KO and control cultures (Fig. 1H-I). Given that IAV exhibits strain-dependent cellular tropism and replication kinetics (e.g., Roach et al., PNAS 2024; <https://doi.org/10.1073/pnas.2320303121>), demonstrating unbiased gene editing across cell populations would strengthen the conclusions. While technically challenging (requiring scRNA-seq or RNA-scope), acknowledging this limitation in the discussion would be sufficient.

We appreciate the reviewer’s comment regarding residual MAVS expression and the lack of cell-type–specific gene editing. We agree that while western blot analysis demonstrates substantial reduction of MAVS, low levels of MAVS expression remain detectable. As noted by the reviewer, we did not directly assess editing efficiency across individual epithelial cell subtypes. Although we confirm the presence of ciliated and secretory populations in both KO and control cultures, unbiased quantification of gene disruption at single-cell resolution was beyond the scope of the present study. We agree that such analyses would further strengthen conclusions regarding cell-type–specific effects and have now acknowledged this as a limitation in the Discussion section.

“While MAVS protein levels were markedly reduced in the MAVS KO cultures, low residual expression remained detectable by western blot. HAE-ALI cultures are heterogeneous, and electroporation may not affect all cells, making complete MAVS elimination challenging. Nonetheless, MAVS protein levels were consistently reduced in KO cultures from both donors compared to controls, allowing investigation of the role of MAVS in this respiratory epithelium model. An important limitation of this study is that, although both ciliated and secretory epithelial populations were confirmed in KO and control cultures, gene editing efficiency was not assessed at single-cell resolution. Given that influenza A virus can exhibit strain-dependent cellular tropism, editing efficiency may vary across epithelial subtypes. Future studies employing single-cell transcriptomic approaches will be valuable in determining the extent of MAVS disruption within distinct epithelial populations.” (page 5, line 6-15).

The claim that MAVS is critical for restricting viral replication is somewhat less compelling. While increased viral gene transcripts and reduced NS1 protein by western blot support this, the immunofluorescence images in Fig. 3E-F are difficult to interpret at the current magnification and resolution. Co-staining for secretory and ciliated cells would help assess tropism and epithelial integrity qualitatively. Additionally, although the data indicate increased viral replication, the most rigorous confirmatory assay would be a plaque-forming assay. Inclusion of discussion acknowledging this limitation would address concerns regarding replication kinetics without PFU measurement.

We thank the reviewer for their comment regarding the assessment of viral replication. We agree that plaque-forming assays provide the most direct quantification of infectious viral particles. In the present study, increased viral gene transcripts together with altered NS1 protein levels were used as indicators of enhanced viral burden in MAVS KO cultures. While this support increased viral replication, we acknowledge that infectious viral titers were not directly measured. We have now added a statement to the Discussion recognizing this limitation.

Regarding the immunofluorescence images, we agree that higher magnification and co-staining for epithelial subtypes would provide additional insight into cellular tropism and epithelial integrity. This has been noted as a limitation and an area for future investigation.

“Although increased viral transcripts and altered viral protein levels in MAVS KO cultures support enhanced viral burden, infectious viral titers were not directly quantified by plaque-forming assay, which remains the gold standard for measuring replication kinetics. Therefore, while our data indicate increased viral gene expression in the absence of MAVS, the extent to which this corresponds to increased production of infectious virions warrants further investigation. In addition, higher-resolution imaging with co-staining of epithelial subtypes would provide greater insight into potential strain-dependent tropism and epithelial integrity following infection.” (page 4, line 44-46 and page 5, line 1-4).

Minor comments:

Increase legibility of the scale bar in Fig. 3E.

The size of the scale bars in all images of Fig. 3E has been increased to increase readability.

Typographical corrections:

"some HAE-ALI cell that wasn't affected by the KO" → "some HAE-ALI cells weren't affected by the KO"

"ProLong Glas Antifade" → "ProLong Glass Antifade"

All minor comments were adjusted in our manuscript.

Reviewer #3 (Significance (Required)):

The core strength and novelty of this study lies in the use of CRISPR-Cas9-mediated knockdown of a gene of interest in a human epithelial system. The authors demonstrate effective gene editing across multiple donors to interrogate a specific pathway. Although the results are limited to the innate responses of the respiratory epithelium-unlike prior studies using murine models, which capture a broader range of cell types and immune responses-the paper provides valuable mechanistic insight into the role of MAVS in the respiratory epithelium, the first line of defense against viral pathogens.

We sincerely appreciate the thoughtful evaluation of our work by Reviewer 3 and their recognition of its strengths. We are particularly pleased that the reviewer highlighted the novelty of the CRISPR-Cas9–mediated gene editing approach in primary human epithelium and acknowledged the value of validating the model across multiple donors. We also appreciate their recognition that, despite the focused scope on epithelial innate responses, the study provides meaningful mechanistic insight into the role of MAVS at the respiratory barrier.

In addition to revising the manuscript in response to reviewer’s comments, we have also made minor grammatical changes, which are listed below:

1. On the → and their (page 1, line 12)
2. Molecule → protein (page 1, line 13)
3. Test → assess (page 1, line 13)
4. Has → is various experiments have (page 2, line 16)
5. However, there are discrepancies regarding the extent to which MAVS contributes to antiviral defense → However, discrepancies regarding the extent to which MAVS contributes to antiviral defense are still present. (page 2, line 18)
6. Moved this sentence from line 23 to line 21 on page 2: “Only a few studies have examined the importance of MAVS in the antiviral defense against IAV, and these studies report no consistent phenotype 9,16,18,20.”.
7. RNA virus infections → IAV infection (page 2, line 23)
8. Deleted “then” (page 2, line 40).
9. Infection with SeV induced C-X-C Motif Chemokine Ligand 10 (CXCL-10) and Interferon Stimulated Gene 15 (ISG-15), measured by ELISA and qPCR, however this induction was decreased in the MAVS KO cultures compared to the control (Fig. 1D, E) → Infection with SeV increased production of C-X-C Motif Chemokine Ligand 10 (CXCL-10) and Interferon Stimulated Gene 15 (ISG-15), measured by ELISA and qPCR. However, this induction was decreased in the MAVS KO cultures compared to the control (Fig. 1D, E), (page 3, line 2-5).
10. Deleted “generated” (page 3, line 12).
11. However → but (page 3, line 16)
12. Was → were (page 3, line 31)
13. Deleted “by qPCR” (page 3, line 32).
14. To examine this, viral RNA corresponding to the segments NP, M2, and NS1 of IAV from infected AAVS1 or MAVS KO HAE-ALI was quantified by qPCR. → To examine this, viral RNA corresponding to the Nucleoprotein (NP), Matrix protein 2 (M2), and Non-structural protein 1 (NS1) of IAV from infected AAVS1 or MAVS KO HAE-ALI was quantified by qPCR. (page 4, line 3-5)
15. Added “the” (page 4, line 25)
16. The current study only includes two same sex donors → A limitation of the current study is the inclusion of only two same-sex donors (page 5, line 16-17)
17. The HAE-ALI model only includes respiratory epithelial cells, and therefore it does not... → Another limitation is, that the HAE-ALI model only includes respiratory epithelial cells. Therefore, it does not … (page 5, line 18-19)
18. Role → importance (page 5, line 20)
19. Importance → role (page 5, line 21)
20. Deleted “the” (page 5, line 21)
21. An important strength of this study is, that it was conducted on primary human respiratory epithelium cells, making the results transferable to humans. → Despite these limitations, an important strength of this study is, that it was conducted on primary human respiratory epithelium cells, making the results applicable to humans. (page 5, line 27-28).
22. Added “Although further research, is needed, this study demonstrates that MAVS KO significantly impairs IFN and ISG induction in a human respiratory epithelial model infected with IAV. Given the physiological relevance of this system, these results support an important role for MAVS in antiviral responses in the human respiratory epithelium.” (page 5, line 34-37).
23. Figure texts: IAV (diluted 1:20 in DMEM) → IAV (MOI 0.5).
